# João Aprígio Guerra de Almeida: supporting breastfeeding mothers

**DOI:** 10.2471/BLT.20.030620

**Published:** 2020-06-01

## Abstract

João Aprígio Guerra de Almeida speaks to Andréia Azevedo Soares about the origins of Brazil’s human milk bank network and the psychosocial aspects of breastfeeding.

Q: How did you become interested in breastfeeding?

A: It started out with an interest in milk. I met the director of a large Brazilian dairy company at a barbecue and was talking to him about my passion for nuclear chemistry and he tried to persuade me to switch to milk research. He said milk is this wonderful product; you can make anything with it – from billiard balls to the glue for airplane wings. This piqued my curiosity and I visited his company where I discussed milk research with a professor. I went on to do a food engineering degree at the Federal University of Viçosa and became very interested in analysing the chemical composition of milk in different mammals.

Q: How did you go from that to focusing on human beings?

A: An obstetrician got in touch with me while I was at university. He was working in a rural area and was trying to establish human milk banks to support breastfeeding mothers. But he was having problems preserving the milk and he asked if I could help. So, I visited two breast-milk banks, and was shocked by the lack of quality control. I actually wrote a letter to the Ministry of Health expressing my concern and decided to refocus my research on the safe collection and preservation of human breast milk.

Q: Did you ever receive an answer from the health ministry?

A: I did. I even got an invitation to meet with some breastfeeding specialists in Rio de Janeiro. I remember sitting in silence until they started talking about microbiology and proposing solutions that didn’t seem right to me. So, I shared some of the findings of the research I had been reading and they eventually invited me to join the national breastfeeding promotion programme as a consultant.

Q: Why is breastfeeding important?

A: The consumption of human milk is one of the most cost-effective interventions we have to support child nutrition. It has been shown to not only promote cognitive development but to boost immune response. One of the best ways to make sure newborns get the breast milk they need is to support the mother’s lactation, but when that’s not enough, or when the mother cannot lactate, human milk banks can play a vital supporting role.

Q: When did you first start working on human milk banks?

A: When I arrived at the Oswaldo Cruz Foundation (Fiocruz) in Rio in 1985 as a visiting researcher. I did an assessment of quality in the foundation’s milk bank and was asked to help improve it. I ended up doing my masters on human milk preservation techniques at Viçosa at the same time as I was redesigning the milk preservation process, establishing quality control systems, and training employees at Fiocruz. I defended my thesis in 1986 and, to show my gratitude to the Fiocruz team, provided them with a proposal to transform their human milk bank into a human milk research centre. To my surprise they called and told me that my proposal had been approved and they invited me back to implement it. At that point I moved to Rio with my family and begun work setting up the research centre in the Fernandes Figueira Institute.

“The biology of milk production is influenced by environmental factors.”

Q: What challenges did you face in setting up the research centre?

A: One of the big challenges for me was embracing the psychosocial aspects of breastfeeding. My focus at that time was on milk, milk, milk. It seemed to me that there was no point in having a cutting-edge lab to ensure the quality and safety of the product if I didn't have the product. So, obviously, I needed some way to engage with the women who could donate. And as ridiculous as it may seem, the female dimension was completely new for me. I was lucky enough to meet the researcher Maria Cecília de Souza Minayo, who guided me towards important bibliographic sources coming from the sociology of health, comparative anthropology and social psychology. Those sources really changed my thinking.

Q: In what way?

A: They made me realize that the breast milk issue cannot be reduced to the simple logistics of milk production and distribution. I came to see that the woman is not simply the means of production– she is a woman who happens to be a mother, who may or may not have chosen motherhood, may or may not have chosen to breastfeed, and who probably has many other responsibilities besides breastfeeding. I came to appreciate the extent to which the biology of milk production is influenced by environmental factors.

Q: How did that change your thinking?

A: It became easier for me to understand why women respond more or less successfully to the role they are presented with. I also saw the extent to which mothers had been marginalized in the paternalistic discourse that had developed around breastfeeding. This can be dated back to the first Brazilian medical thesis focused on the topic, which was presented in 1838 and titled, *The usefulness of breastfeeding and the inconveniences that result from the disrespect of such a duty*. That ‘dereliction of duty’ theme finds echoes in subsequent research linking weaning to workforce participation.

Q: Can you say more about that?

A: It has become received wisdom that Brazil’s weaning ‘epidemics’ of the 1970s and 1990s were linked to increased urbanization of the population and the entry of women into the labour market (along with aggressive marketing of industrially manufactured infant formula). However, when my colleagues and I conducted a qualitative survey of women living in different regions of Brazil to discover their reasons for weaning, the main reasons given were "low milk supply" and "milk dried up". We found no correlation between weaning and participation in formal employment. That suggests to me that we should not be so quick to link employment to weaning.

Q: How did your research change your approach to supporting breastfeeding mothers?

A: As I said, when I started out, my primary concern was the milk, how to get sufficient quantities of milk to support breastfeeding mothers. After doing the research, I came to the view that this focus on milk as a commodity and women as producers risked perpetuating some of the unhelpful trends revealed in my reading. My colleagues and I felt very strongly that we should not allow our milk banks to be transformed into human dairies. For us the protagonist in the breastfeeding story is the woman and not her milk or even her child. Our core concern became how best to support women so that they could breastfeed their own children.

“We should not be so quick to link employment to weaning.”

Q: How is this change of approach expressed in the way your centres are organized?

A: The primary function of people working in the support centres is to bond with women, to listen without judgement, so that women can tell their own stories, and to build trust. It is when that trust has been created that medical advice about all the issues that can occur during breastfeeding and how to breastfeed according to WHO guidelines can be most effectively delivered. Above all, it is only after achieving a bond with the women that we think about collecting, testing, pasteurizing and distributing milk. The idea is to build a large breastfeeding community, which then generates the milk donations, which can be used to help mothers when required.

Q: Collecting, testing, pasteurizing and distributing milk are important functions?

A: Absolutely, and we take great pride in our capacity to perform those functions cost effectively. But I stress the importance of supporting mothers because it is easily overlooked. And that includes reaching out beyond the walls of our facilities to support women in the community. So that the mother who calls our toll-free helpline at three o’clock in the morning, a mother whose baby is crying and who may be utterly stressed out because of the drug-trafficking and shooting that is happening outside her apartment, can find a qualified person to listen to her. Having that possibility changes the mother's experience. She is no longer alone with her child. She is supported.

Q: Today Brazil’s human milk network comprises 224 human milk banks and 215 collection centres. What has been the impact of the initiative on breastfeeding in Brazil?

A: It has been credited with a substantial increase in breastfeeding and a fall in infant mortality. For example, the prevalence of exclusive breastfeeding in children under six months of age increased from 4.7% in 1986 to 37.1% in 2006. However, it has since levelled off and more needs to be done to keep trends positive. It is also important to recognize that the initiative is one of several, including the National Breastfeeding Programme created in 1981, which introduced regulation of the commercialization of infant food, implementation of the Child Friendly Hospitals policy, the implementation of the Brazilian Breastfeeding and Complementary Feeding Strategy, and, most recently, efforts to support working women who breastfeed.

Q: You have actively supported similar human milk initiatives in other countries. What drove that initiative?

*A: *The Sasakawa Health Prize in 2001 increased the visibility of our work and led to requests for international technical cooperation. In 2005 we decide to organize this cooperation and started working with other Latin American countries, which did not have human milk banks at that time. The few that existed had been closed in the 1980s, including in Central America because of concerns about transmission of HIV through breast milk. Now we work together not only with countries in South and Central America, but also with Portugal and Spain, and Portuguese-speaking African countries.

Q: How does the approach adapt to different settings?

A: It has been implemented across a range of setting without problems, largely because we had already adapted the model to suit the different conditions encountered in Brazil. Also, it is important to note that we do not reproduce or transfer the human milk bank model; rather, we transmit the principles that underlie our model and at the core of those principals is the vital importance of supporting the mother.

